# Association of *PON1-L55M* Genetic Variation and Breast Cancer Risk: A Case-Control Trial

**DOI:** 10.31557/APJCP.2020.21.1.255

**Published:** 2020

**Authors:** Amir Farmohammadi, Ali Momeni, Banafshe Bahmani, Hossein Ghorbani, Ramin Ramzanpour

**Affiliations:** 1 *Student Research Committee, *; 2 *School of Medicine, Isfahan University of Medical Sciences, Isfahan, *; 3 *Department of Pathology, Faculty of Medicine, Kashan University of Medical Sciences, Kashan, *; 4 *Pathology Department, *; 5 *Cellular and Molecular Biology Research Center, Health Research Institute, Babol University of Medical Sciences, Babol, Iran. *

**Keywords:** Breast cancer, paraoxonase 1, genetic polymorphism, PCR-RFLP

## Abstract

**Background:**

Paraoxonase 1 (PON1), a multifactorial antioxidant enzyme, has a defensive role against oxidative stress, which is believed to contribute to cancer development. This study aimed to investigate the association of PON1-L55M functional polymorphism with breast cancer risk.

**Material and methods:**

In the experimental study, blood samples were collected from 150 healthy women controls and 150 breast cancer subjects. The L55M genotyping was performed by polymerase chain reaction-restriction fragment length polymorphism.

**Results:**

Our analysis showed that the genotypes distribution is in Hardy-Weinberg equilibrium for both case and control groups. Our data revealed that there are significant associations between *PON1-L55M* polymorphism and breast cancer risk in homozygote (OR= 2.13, 95%CI= 1.14-4.00, p= 0.018), dominant (OR= 1.72, 95%CI= 1.07-2.76, p= 0.024), and allelic (OR= 1.55, 95%CI= 1.12-2.15, p= 0.008) models.

**Conclusions:**

Our results suggest that the *PON1-L55M* genetic variation could be a genetic risk factor for breast cancer risk and it could be considered as a molecular biomarker for screening of susceptible women.

## Introduction

Breast cancer (BC) in women is the most common reason for cancer mortality around the world. The rate of this malignancy is varying worldwide, but it is increasing in areas that until recently had a low prevalence of the disease (Key et al., 2001). Many etiologic factors, including nutritional, lifestyle, environmental carcinogenicity, as well as oxidative stress have been considered to increase the risk of breast cancer (Calaf et al., 2018; Wu et al., 2018). The role of paraoxonase 1 (*PON1*) in detoxification of cancer-causing oxidative stress encourages scientists to evaluate *PON1* gene variations in susceptibility to breast cancer (Pan et al., 2019). *PON1* binds to HDL and helps to detoxify organophosphorus compounds such as paraoxon and lipid peroxidation-soluble radicals (Shih et al., 1998).

Humans *PON1* gene is located on the long arm of chromosome 7 (7q21.3) which is contained 9 exons and encodes a protein with 355 residues (Deakin et al., 2002). Although several pharmaceutical, dietary, and life-style modulators of *PON1* are identified, by far the main influence on the activity of* PON1*, which could differ by above 40 times between persons, is through *PON1* genetic variations (Costa et al., 2005; Mackness and Mackness, 2015). There are several main polymorphisms in *PON1* gene that could affect the gene function. The* PON1-Q192R (rs662)* missense single nucleotide polymorphism (SNP) regulates a substrate-dependent influence on activity. The 192R isoform could hydrolyze some substrates such as paraoxon 192Q isoform could hydrolyze some other substrates such as lipid-peroxides and diazoxon (Mackness et al., 1998). Both the missense L55M (rs854560) and the upstream T-108C (rs705379) SNPs are correlated with various activities and serum levels of *PON1*. The PON1-55M isoform leads to a lower level of mRNA and then its activity compared to 55L isoform. Besides, the -108C genotype of rs705379 variation could result in higher promoter activity compared to the -108T genotype (Costa et al., 2005; Schrader et al., 2012). There are several studies investigating the role of three mentioned SNPs with various common malignancies e.g. breast cancer. Regarding to the rs854560 polymorphism, there are some reports. For example, Stevens et al., (2006) reported that this polymorphism might be correlated with elevated susceptibility of breast cancer in USA. Naidu et al., (2010) suggested that the L55M SNP may be a genetic biomarker for breast cancer in Malaysian population. However, there is no identified study investigating the association of this polymorphism with breast cancer in Iranian population. This study aimed to investigate the association of *PON1-L55M* genetic polymorphism with breast cancer susceptibility in a case-control trial.

## Materials and Methods

*Subjects*

In a hospital-based case-control study, 300 participants including 150 subjects with breast cancer and 150 age-matched healthy controls were enrolled from Pasteur pathobiology and genetics laboratory and also Rohani hospital (Babol, Iran). The breast cancer diagnosis was approved histologically for all breast cancer subjects. Healthy controls individuals without oncological history were referring to the same hospital for routine tests, and after completing the questionnaire and oral interview, it became clear that they did not display positive outcomes. Lastly, 2 mL peripheral blood was obtained from all participants. Besides, the current study was performed according to the criteria outlined in the Helsinki Declaration. 

*SNP genotyping*

After, blood sample collection, genomic DNA was extracted from whole blood by a commercial Kit (Bioneer, Korea) and the *PON1-L55M SNP* genotyping was performed by polymerase chain reaction-restriction fragment length polymorphism (PCR-RFLP) method according to the previous study (Kocakap et al., 2016). In brief, PCR was done in a 20 µL overall volume. About 50 ng of genomic DNA, 10 µL of 2X premix, and 0.1 nmoL of the primers (forward primer: 5’-GAAGAGTGATGTATAGCCCCAG-3’ and reverse primer: 5’-TTTAATCCAGAGCTAATGAAAGCC-3’) were used to PCR. After initial denaturation at 95°C for 5 min, the PCR mixture was subjected to 30 repeatitive cycles of denaturation (at 94°C for 1 min), annealing (at 61°C 45 sec), and extension (at 72°C for 1 min). The PCR products with length of 171-bp was treated with the NlaIII restriction endonuclease (Fermentas, Germany) at 37°C for 16 hours, and the digested mixtures were electrophoresed on 2% agarose gel. The T (L) allele had no restriction site for NlaIII enzyme, while the A (M) allele was digested to 127 and 44-bp fragments.

*Statistical analysis*

Hardy-Weinberg equilibrium (HWE) was computed in both case and control groups by the Chi-Square test. The differences of allele and genotype frequencies between case and control groups were calculated by Chi-Square test. The strength of association between *rs854560 *polymorphism and risk of breast cancer was evaluated by odds ratios (ORs) and 95% confidence intervals (CIs) which were estimated by logistic regression. A p-value of less than 0.05 was considered statistically significant. These statistical analyses were performed by SPSS Statistical software version 19 (SPSS Inc., Chicago, IL, USA).

## Results

Some demographic and demographic features of included patients in our study are presented in table 1. The average of age and body mass index (BMI) were estimated 45.50±7.26 years and 25.10±4.15 kg/m^2^, respectively. Additionally, 41.33% of patients were postmenopausal. The highest percentage of histologic grade was related to grade I (52.00%). The tumor type for 67.33% of patients was detected as invasive ductal carcinoma (IDC) and 32.00% was detected as invasive lobular carcinoma (ILC). While one of the patients was considered with a mixed tumor (IDC+ILC). Tumor size in 65.33% of patients was less than 2 cm while 34.67% of patients have a tumor with size ≥2 cm. Finally, lymph node metastasis was observed in 52.00% of patients (Table 1).

Genotype frequencies of *PON1-rs854560* variation were computed and data examination showed that genotypes distribution met the HWE criteria in patient (χ^2^= 2.54; *p= 0.111*) and control (χ^2^= 3.37;* p= 0.067*) groups. Table 2 displays frequencies of allele and genotype for the *rs854560 SNP* in our case-control study. Frequencies of genotypes LL, LM, and MM were 44.00%, 39.33%, and 16.67%, respectively for the healthy control group and 31.33%, 43.33%, and 25.34% respectively for the breast cancer group. Alleles L and M rates were 63.67% and 36.33% respectively for the control group and 53.00% and 47.00% respectively in the breast cancer group. As detailed in Table 2, we observed a significant association between genotype MM and breast cancer risk (OR= 2.13, 95%CI= 1.14-4.00, *p= 0.018*). In addition, carriers of allele M were at a high risk for breast cancer (OR= 1.72, 95%CI= 1.07-2.76, *p= 0.024*). Besides, allele analysis revealed that there is a significant association between allele M and breast cancer risk (OR= 1.55, 95%CI= 1.12-2.15, *p= 0.008*).

**Table 1:** Demographic and Pathologic Information of Patients

Variables	Description
Age (years) mean±SD	45.50±7.26
BMI (kg/m^2^) mean±SD	25.10±4.15
Menopause status	
Yes	62 (41.33%)
No	88 (58.67%)
Histological grade	
I	78 (52.00%)
II	29 (19.33%)
III	13 (08.67%)
Not identified	30 (20.00%)
Tumor type	
IDC	101 (67.33%)
ILC	48 (32.00%)
IDC+ILC	1 (0.67%)
Tumor size	
<2 cm	98 (65.33%)
≥2 cm	52 (34.67%)
Lymph node metastasis	
No	72 (48.00%)
Yes	78 (52.00%)

BMI, body mass index; SD, standard deviation; IDC, invasive ductal carcinoma; ILC, invasive lobular carcinoma

**Table 2:** Genotype and Allele Frequensies of rs854560

Genotype/ Allele	No. and Percentage		Chi-square	OR (95% CI)	p-value
	Control (n= 150)	Case (n= 150)			
LL	66 (44.00%)	47 (31.33%)	-	-	-
LM	59 (39.33%)	65 (43.33%)	2.78	1.55 (0.93-2.59)	0.096
MM	25 (16.67%)	38 (25.34%)	5.68	2.13 (1.14-4.00)	0.018
LM+MM	84 (56.00%)	103 (68.67%)	5.13	1.72 (1.07-2.76)	0.024
L	191 (63.67%)	159 (53.00%)	-	-	-
M	109 (36.33%)	141 (47.00%)	7.02	1.55 (1.12-2.15)	0.008

OR, odds ratio; CI, confidence interval; Significant differences between the case and control groups are bolded.

## Discussion

Breast cancer is the most common cancer in women that the etiology of this malignancy is not fully understood. It seems that oxidative stress and free radicals play an important role in the pathogenesis and progression of breast cancer (Valko et al., 2006; Hecht et al., 2016). In this study, we investigated the association of the common polymorphism L55M in the* PON1* antioxidant gene in a case-control study. The data from our experimental study revealed that there is a significant association between MM, LM+MM, M genotypes and allele and breast cancer risk in our studied population. This shows the main role of this gene in the development of breast cancer. In a meta-analysis published in 2019, it was reported that there are significant associations between PON1-L55M genetic variation and other cancers such as hematologic cancer and prostate cancer (Pan et al., 2019). However, there is another key polymorphism in the *PON1* gene which should be noted. It is Q192R missense variation which is associated with decreased risk of breast cancer (Pan et al., 2019). 

Oxidative stress could result in impaired in biological membranes, intracellular organelles, and macromolecules such as proteins and DNA (Essick and Sam, 2010). One of the main damage is the oxidation of lipids by free radicals resulting from these stresses, which results in the production of active compounds such as aldehydes, ketones and hydroxyl acids (Valko et al., 2006). These radicals may have an external source or may be due to oxidation-reduction reactions in the body. The imbalance in the formation and removal of these free radicals, including reactive oxygen species (ROS), has been shown to cause genetic dysfunction, interference with cellular signaling, metastasis, neurodegenerative diseases, and aging (Allen, 1998). The human body has many enzymatic systems for the protection of genotoxic damage that activate, indirectly, through the reduction of substrates having the potential to produce free radicals, such as cytochrome P450c17a or directly via or indirectly, through free radical scavenging, such as *PON1* (Yu, 1994; Salama et al., 2008). Therefore, it is accepted that the polymorphisms of these genes are key determinants of cancer susceptibility to toxic or environmental toxic chemicals (Shih et al., 1998; de Jong et al., 2002). Single nucleotide polymorphisms based on their gene locations could affect the function of the gene (Salimi et al., 2017; Nejati et al., 2018). For example, genetic variations in promoter regions could affect gene expression level and non-coding variation could alter gene expression via interfering with splicing procedure (Mobasseri et al., 2019; Zamani-Badi et al., 2019). But, the genetic variations in coding sequences (exon variations) could affect the protein structure and function (Noureddini et al., 2018; Bafrani et al., 2019). So based on what was mentioned, the L55M polymorphism as an exon variation, could affect the protein *PON1* structure and/or function. Investigation of molecular effects of genetic variation through in vitro and in vivo is too cost- and time-consuming. However, the computational analysis could be useful to evaluate these effects and other molecular evaluations (Tameh et al., 2018; Zamani-Badi et al., 2018). Therefore, assessment of L55M polymorphism on the PON1 protein function by using bioinformatics tools could be a helpful approach. 

Our findings suggest that the *PON1-L55M* polymorphism is a genetic risk factor for breast cancer risk and it could be considered as a possible molecular biomarker for screening of susceptible women. However, there are some limitations in case-control study which should be mentioned. For example, we did not evaluate the gene-gene and gene-environmental factors and also our sample size was somewhat small. Besides, combination effects of *PON1-L55M* gene polymorphism and some oxidative stress markers such as total antioxidant capacity, malondialdehyde with breast cancer could be useful.

## References

[B1] Allen RG (1998). Oxidative stress and superoxide dismutase in development, aging and gene regulation. Age.

[B2] Bafrani HH, Ahmadi M, Jahantigh D (2019). Association analysis of the common varieties of IL17A and IL17F genes with the risk of knee osteoarthritis. J Cell Biochem.

[B3] Calaf GM, Urzua U, Termini L (2018). Oxidative stress in female cancers. Oncotarget.

[B4] Costa LG, Vitalone A, Cole TB (2005). Modulation of paraoxonase (PON1) activity. Biochem Pharmacol.

[B5] de Jong MM, Nolte IM, te Meerman GJ (2002). Low-penetrance genes and their involvement in colorectal cancer susceptibility. Cancer Epidemiol Biomarkers Prev.

[B6] Deakin S, Leviev I, Gomaraschi M (2002). Enzymatically active paraoxonase-1 is located at the external membrane of producing cells and released by a high affinity, saturable, desorption mechanism. J Biol Chem.

[B7] Essick EE, Sam F (2010). Oxidative stress and autophagy in cardiac disease, neurological disorders, aging and cancer. Oxid Med Cell Longev.

[B8] Hecht F, Pessoa CF, Gentile LB (2016). The role of oxidative stress on breast cancer development and therapy. Tumour Biol.

[B9] Key TJ, Verkasalo PK, Banks E (2001). Epidemiology of breast cancer. Lancet Oncol.

[B10] Kocakap DBS, Doğru MT, Şimşek V (2016). The association of paraoxonase 1 gene L55M polymorphism with the extent and severity of coronary artery disease in the Turkish population and its dependence on gender. Anatol J Cardiol.

[B11] Mackness B, Durrington PN, Mackness MI (1998). Human serum paraoxonase. General Pharmacology: Gen Pharmacol.

[B12] Mackness M, Mackness B (2015). Human paraoxonase-1 (PON1): Gene structure and expression, promiscuous activities and multiple physiological roles. Gene.

[B13] Mobasseri N, Nikzad H, Karimian M (2019). Protective effect of oestrogen receptor α-PvuII transition against idiopathic male infertility: a case-control study and meta-analysis. Reprod Biomed Online.

[B14] Naidu R, Har YC, Taib NAM (2010). Genetic polymorphisms of paraoxonase 1 (PON1) gene: association between L55M or Q192R with breast cancer risk and clinico-pathological parameters. Pathol Oncol Res.

[B15] Nejati M, Atlasi MA, Karimian M (2018). Lipoprotein lipase gene polymorphisms as risk factors for stroke: a computational and meta-analysis. Iran J Basic Med Sci.

[B16] Noureddini M, Mobasseri N, Karimian M (2018). Arg399Gln substitution in XRCC1 as a prognostic and predictive biomarker for prostate cancer: Evidence from 8662 subjects and a structural analysis. J Gene Med.

[B17] Pan X, Huang L, Li M (2019). The association between PON1 (Q192R and L55M) gene polymorphisms and risk of cancer: A meta-analysis based on 43 studies. Biomed Res Int.

[B18] Salama SA, Kamel M, Awad M (2008). Catecholestrogens induce oxidative stress and malignant transformation in human endometrial glandular cells: Protective effect of catechol-O-methyltransferase. Int J Cancer.

[B19] Salimi S, Keshavarzi F, Mohammadpour-Gharehbagh A (2017). Polymorphisms of the folate metabolizing enzymes: Association with SLE susceptibility and in silico analysis. Gene.

[B20] Schrader C, Graeser AC, Huebbe P (2012). Allyl isothiocyanate as a potential inducer of paraoxonase-1-studies in cultured hepatocytes and in mice. IUBMB Life.

[B21] Shih DM, Gu L, Xia Y-R (1998). Mice lacking serum paraoxonase are susceptible to organophosphate toxicity and atherosclerosis. Nature.

[B22] Stevens VL, Rodriguez C, Pavluck AL (2006). Association of polymorphisms in the paraoxonase 1 gene with breast cancer incidence in the CPS-II Nutrition Cohort. Cancer Epidemiol Biomarkers Prev.

[B23] Tameh AA, Karimian M, Zare-Dehghanani Z (2018). Role of steroid therapy after ischemic stroke by N-methyl-d-aspartate receptor gene regulation. J Stroke Cerebrovasc Dis.

[B24] Valko M, Rhodes C, Moncol J (2006). Free radicals, metals and antioxidants in oxidative stress-induced cancer. Chem Biol Interact.

[B25] Wu S, Zhu W, Thompson P (2018). Evaluating intrinsic and non-intrinsic cancer risk factors. Nat Commun.

[B26] Yu BP (1994). Cellular defenses against damage from reactive oxygen species. Physiol Rev.

[B27] Zamani-Badi T, Karimian M, Azami-Tameh A (2019). Association of C3953T transition in interleukin 1β gene with idiopathic male infertility in an Iranian population. Hum Fertil.

[B28] Zamani-Badi T, Nikzad H, Karimian M (2018). IL-1RA VNTR and IL-1α 4845G> T polymorphisms and risk of idiopathic male infertility in Iranian men: A case–control study and an in silico analysis. Andrologia.

